# A Remote Sensor for Detecting Methane Based on Palladium-Decorated Single Walled Carbon Nanotubes

**DOI:** 10.3390/s130708814

**Published:** 2013-07-10

**Authors:** Jian Liu, Guomin Li

**Affiliations:** School of Communication and Information Engineering, Xi'an University of Science and Technology, No.58, Yan Ta Road, Xi'an 710054, China; E-Mail: liguomin@xust.edu.cn

**Keywords:** gas sensor, methane concentration, Pd-SWCNTs, RFID, room temperature

## Abstract

The remote detection of the concentration of methane at room temperature is performed by a sensor that is configured by the combination of radio frequency identification (RFID), and functionalized carbon nanotubes (CNTs). The proposed sensor is schemed as a thin film RFID tag in a polyethylene substrate, on which a metal trace dipole, a metal trace T impedance matching networks, a 0.5 μm-CMOS RF/DC rectifier chipset and a sensor head of palladium-decorated single walled carbon nanotubes (Pd-SWCNTs) are surface mounted in cascade. The performances of the sensor are examined and described by the defined parameters of the received signal strength index (RSSI) and the comparative analog identifier (ΔAID). Results validate the sensor's ability to detect molecules of methane at room temperature, showing that the RSSI can increase 4 dB and the ΔAID can increase 3% in response to methane concentrations ranging from zero to 100 ppm.

## 1. Introduction

The risk of explosion is the primary concern for accurate measurement the methane because a concentration level as low as 4% in air can become explosive [1]. This is the reason why in hazardous environments like coal mines, the measurement of methane is necessary; and remote measurement, is a more appropriate method, even if it is a complicated case, dealing with not only the sensing mechanism, the wireless protocol, but also the combination of technologies for performance trade-offs [2]. Although the commercially available catalytic beads and metal oxides are regarded as the most suitable technology for detecting methane concentrations of hundreds of parts per million (ppm), and the infrared and flame ionization techniques are especially suited for instrumental-level analysis for methane plus other hydrocarbons over a range from sub-parts per million (ppm) to parts per billion (ppb), they cannot be easily implemented for the most ordinary environments because of their use of sensing mechanisms involving either chemical oxidation or methane decomposition that have been verified to consume a large quantity of energy, and the required temperatures are usually high, up to 500 °C. It is thus evident that study of sensors that are capable of detecting the concentration of methane at room temperature air is quite necessary [3,4].

Since the emergency of nanotechnology in 1991, rapid progress in the synthesis and fundamental understanding of surface phenomena has generated a great deal of excitement about the incorporation of nanomaterials into gas sensor architectures. For example, carbon nanotubes (CNTs), are, in principle, one-dimensional molecular structures obtained by rolling up one graphene sheet into one seamless cylinder producing single-wall-carbon-nanotubes (SWCNTs), or instead, into more cylinders to afford multi-wall-carbon-nanotubes (MWCNTs) [5]. As a result, some certain types of CNTs are composed almost entirely of surface atoms, reducing their dimensions to the order of several nanometers can give surface chemistry events a more important role than in the bulk state, whereby the reduced dimensionality will create structures with exceptionally high surface areas, creating an increase in environmental sensitivity [6]. Therefore, CNTs facilitate the fabrication of environmental sensors so as to make easy gas detection feasible.

This property can really make CNTs become gas sensors by following different working principles that involve miniaturized ionizing gas sensors (also called “micro-gun” sensors) [7], thin films of CNTs with variable resistance as a function of the adsorbed gas properties [8-11], and carbon nanotube field effect transistors (CNTFETs) as well. Taking the CNTFET-based gas sensors into consideration, research is exclusively focused on SWCNTs (which can be semiconducting or metallic, depending on their chirality [12]) because the MWCNTs are only metallic, and therefore unsuitable to fabricate transistors [13].

In addition, because the CNTs' conduction channel is composed almost entirely of surface atoms, and even small changes in the local chemical environment will results in measurable changes in the device conductance, CNTFET-based gas sensors are adeptly at monitoring molecules of gases in ambient air. For example, the semi-conducting CNTs are P-type under ambient conduction [14], so the electron donation into the valence band will result in charge-carrier (h+) recombination, causing a decrease in conductance and a shift in the transfer characteristic curve I-V_G_ towards more negative voltages; the electron withdrawal, conversely, serve to increase the hole concentration in the CNTs, leading to an increase in conductance and a shift in the transfer characteristic I-V_G_ towards more positive voltages.

Evidently, the change of conductance in CNTFETs reflects the change of chemical compounds that is exactly the mechanism used for governing the CNTFET-based sensors to sense the molecules of gases. Nowadays, one can find a few CNTFET-based gas sensors in terms of NO_2_, O_2_, or ; CO, H_2_ and NH_3_, but there are few examples for the inert gases like CH_4_ that are said to display intrinsically low polarity [15-18]. Nonetheless, we can still find a few recently reported studies. The adsorption of CH_4_ onto SWCNTs was calculated to result in a donation of approximately 0.025 electrons per adsorbed molecule, though the experimental confirmation of this conclusion was difficult [19]. The response of palladium (Pd)-decorated SWCNTs toward the concentration of methane were investigated with the resulting curves delineating the change of conductance of Pd-SWCNTs *versus* the CH4 concentration from 6 ppm to 100 ppm in room temperature [20]. CNTs and nanofibers prepared by electrodeposition were shown to result in the dependence of the performance toward CH_4_ upon the concentration index [21]. The deposition of conducting polymers of substituted polythiophenes was explored and it was demonstrated that their impedance varied with the concentration of CH_4_ in room temperature air conditions [22]. However, as pointed by Kauffman and Star, the lack of CNT-based sensors for the detection of CH_4_ is an indication that further study into the fundamental interactions between this gas and functionalized CNTs is a necessity [6].

Besides the sensing mechanism, the remote detection of the concentration of methane depends on the wireless protocols that determine the efficiency and the effectiveness of the transmission of the sensed signal through space. Among the advanced wireless protocols, including sensor networks (SNs), wireless local area networks (WLANs) and radio frequency identification (RFID), RFID is proven to provide advantages such as low cost, light weight, high reliability, energy efficiency and being battery free; the higher sensibility and the longer readable distance make it the best choice even in extreme environments including the enclosed tunnel spaces in coal mines [23-25].

In this regard, this paper attempts to put forward a remote sensor scheme in order to make the detection of methane at room temperature possible. The proposed sensor is composed of a thin film RFID tag in a polyethylene substrate. The function of the remote detection is realized by the integration of several functional modules that are a metal trace dipole, a metal trace T impedance match network, a 0.5 μm-CMOS RF/DC rectifier chipset and a sensor head made from Pd-decorated SWCNTs. The characterization of the sensor is examined, and its performance is described by the parameters of the receiving signal strength index, *i.e.*, RSSI and the comparative analog identifier, *i.e.*, ΔAID that are defined and derived in the paper.

## 2. Principle of Methane Detection Based on the RFID Architecture

In a complicated and hazardous environment, the detection of methane using a RFID architecture usually employs a backscattering scheme for signal delivery [26]. In principle, a reader device sends out a RF signal to a sensor tag to commence a specific detection event by powering the sensor tag till the sensing mechanism is turned on. Afterwards, the sensor tag modulates the sensed information and, simultaneously, backscatters it to the reader device. Once received, the reader device will pass this signal to the integrated circuit (IC), which demodulate and process the signal till the concentration of methane is recognized.

Given the amplitude shift keying (ASK) as the modulation format of the sensor tag. *φ*(*t*) represents the function of the concentration of methane variation with time. For simplicity, the polarization of the antenna in the reader device is assumed to be properly matched to that of the antenna in the sensor tag; so the mismatch coefficient is thus set at 0 dB.

**Figure 1.**
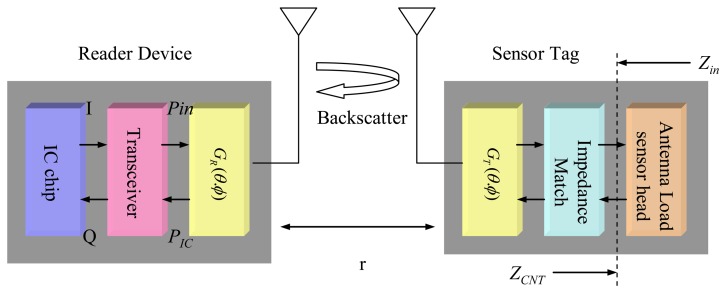
Principle of RFID-based methane detection.

Setting *P_in_* to represent the signal power at the antenna port in reader device that is subjected to be delivered to the sensor tag through space channel. Accordingly, at the antenna port in sensor tag, the signal power *P_CNT_* will be:

(1) $$P_{\text{CNT}}\;(\varphi )={\left(\frac{\lambda_{0}}{4\pi r}\right)}^{2}P_{in}G_{R}\;(\theta ,\phi )G_{T}\;(\theta ,\phi )\tau [\varphi (t)]$$

in which, *λ_0_* represents the wavelength in free space; *r* represents the distance between the reader device and the sensor tag; *G_R_*(*θ*,*ϕ*) represents the antenna gain of the reader device; *G_T_*(*θ*,*ϕ*) represents the antenna gain of the sensor tag that deals with not only the antenna itself, but also the impedance match networks; *τ*[*φ*(*t*) ] is the coefficient of power transmission from the antenna of the sensor tag to the antenna load. The antenna load is a functional module that, in this design, will be made from the Pd decorated SWCNTs that carries out the sensing mechanism engaged for detecting methane in ambient air:

(2) $$\tau [\varphi (t)]=\frac{4R_{\text{CNT}}\;[\varphi (t)]R_{in}}{{\left|Z_{\text{CNT}}\;[\varphi (t)]+Z_{in}\right|}^{2}}$$

where, *Z_CNT_*[*φ*(*t*)] stands for the input impedance toward the antenna load in the sensor tag; and *Z_in_*, conversely, stands for the input impedance toward the antenna in the sensor tag, as illustrated in Figure 1.

The sensor tag works for backscattering the sensed signal to the reader device. *P_IC_* denotes the power of the signal in the reader device that is going to be delivered to the IC chipset for performance recognition:

(3) $$P_{IC}\;(\varphi )={\left(\frac{\lambda_{0}}{4\pi r}\right)}^{4}P_{in}G_{R}^{2}\;(\theta ,\phi )G_{T}^{2}\;(\theta ,\phi )\tau^{\prime }[\varphi (t)]$$

in which, *τ′* [*φ*(*t*) ] is the power transmission coefficient representing the power delivered from the antenna load to the antenna of the sensor tag:

(4) $$\tau^{\prime }[\varphi (t)]={\left(\frac{2R_{in}}{\left|Z_{\text{CNT}}\;[\varphi (t)]+Z_{in}\right|}\right)}^{2}$$

Once received, the reader device will pass *P_IC_* to the IC chip. The IC chip will process the signal till it shows the value of the received signal strength index, *i.e.*, RSSI, a specialized parameter for identifying the concentration of methane that, if in dBm unit, will be expressed as:

(5) $$\text{RSSI}=-43.96+40\text{lg}(\lambda_{0})-40\text{lg}(r)+10\text{lg}(p_{in})+20\text{lg}[G_{R}(\theta ,\phi )]+10\text{lg}\{\tau^{\prime }[\varphi (t)]\}$$

## 3. Fabrication of a Sensor Tag for Methane Detection

**Figure 2.**
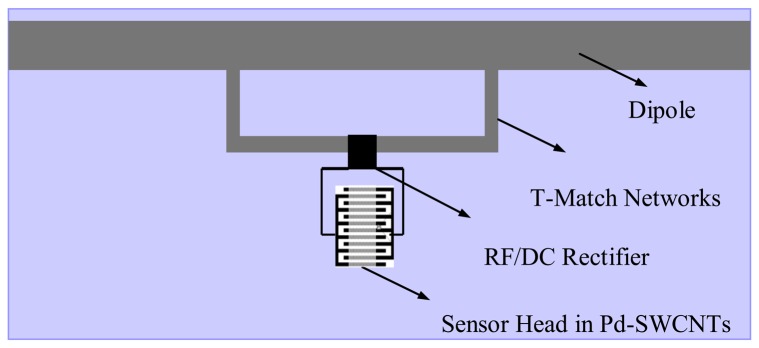
Proposed sensor tag.

According to the RFID-based methane detection scheme described above, the sensor tag accommodates multiple functions to sense, modulate and backscatter the signals carrying the information about the methane levels. A sensor tag, therefore, should have two functional modules: one is the antenna module; the other is the antenna load module. The antenna module sends backscattering and data collection signals to and from the reader device, usually comprising an electromagnetic power radiator and, if necessary, a network for impedance matching; the antenna load senses the methane, and generally contains a sensor head and, if required, a RF/DC rectifier to convert the power collected on the radiator to a DC voltage to replace the role of the externally mounted battery in case of the identification is passively implemented. The proposed sensor tag is illustrated in Figure 2, showing a thin film RFID tag on a polyethylene substrate. The radiator is a metal trace dipole; the impedance match network is a metal trace T structure; the RF/DC rectifier is a 0.5 μm-CMOS chipset; the sensor head is an inter-digitated electrode (IDE) loaded with Pd-decorated SWCNTs. All components in the functional modules are small sized and low profile that therefore amenable for equipment assembly by screen-printing.

### 3.1. Dipole and T Impedance Match Networks

The dipole collects the electromagnetic power from the reader device, and scatters back the sensed signal, so optimal reception is required. The T impedance matching network matches the impedance of the dipole toward the impedance of the antenna load, and *vice versa*, so conjugation is desirable for the delivery of maximum power.

**Figure 3.**
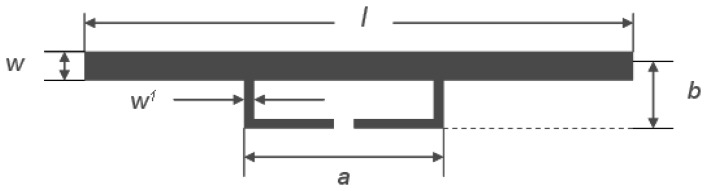
Illustration of the dimensions of the antenna module.

The details of the dimension of the dipole and the T impedance matching network are illustrated in Figure 3. The length of the dipole *l* can be changed by introducing a centered short-circuit stub; the T impedance match networks is configured by a short dipole in the length of *a* ≤ *l* at a close distance of *b*, the input impedance on the port of T impedance match networks can be expressed as:

(6) $$Z_{in}=\frac{2Z_{t}{\left(1+\alpha \right)}^{2}Z_{A}}{2Z_{t}+{\left(1+\alpha \right)}^{2}Z_{A}}$$

where, *Z_t_* = *jZ*_0_ tan *ka*/2 represents the input impedance of the short-circuit stub formed by the T impedance match networks' conductors and the part of the dipole; $Z_{0}≅276\log_{10}\left(b/\sqrt{r_{e}r_{e}^{\prime }}\right)$ represents the characteristic impedance of the two-conductor transmission line with spacing of *b*; *Z_A_* is the dipole impedance taken at the center in absence of T impedance matching network connection. For the metal trace dipole and the metal trace T impedance matching network, *r_e_*= *0.25w* and $r_{e}^{\prime }=8.25w\prime$ are the equivalent radii of the dipole and the matching stub, and $\alpha =\text{In}(b/r_{e}^{\prime })\text{In}(b/r_{e})$ is the current division factor between the two conductors.

### 3.2. Pd-Decorated SWCNTs

The sensor head senses the methane, so it's crucial effect on the overall performance of the sensor tag is certain. Room temperature operation becomes a key issue in this regard. According to the study of Lu *et al.* [20], we chose Pd-decorated SWCNTs that, as a load, will be dispensed on an IDE platform.

**Figure 4.**
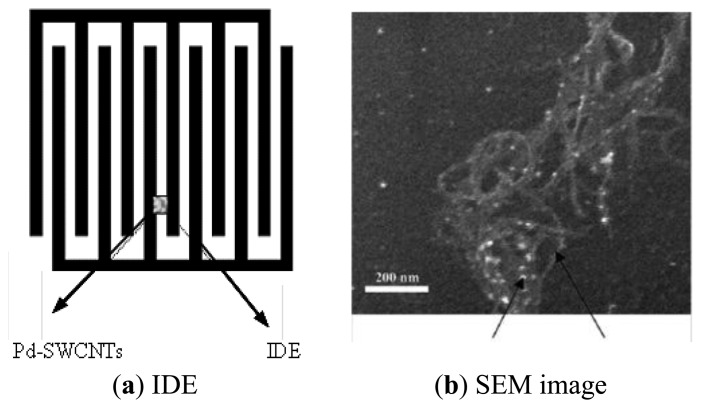
Illustration of (**a**) IDE and (**b**) a SEM image of Pd-decorated SWCNTs. (copyright permission obtained from Elsevier).

The structure of the proposed sensor head is shown in Figure 4. Figure 4a shows the inter-digitated fingers using a finger width of 10 μm and a gap distance of 8 μm; Figure 4b is a scanning electron microscope (SEM) image of a Pd-decorated SWCNT bundle dispersed on the inter-digitated fingers. The bright spots are Pd particles with a measured average size of 10 nm. Pure SWCNTs (98%, Carbon Nanotechnology Inc., Houston, TX, USA) are used as supporting materias. A layer of 10 nm thick metallic Pd was sputter coated onto a pile of SWCNT powder and mixed well with the SWCNTs by shaking [20]. The Pd-decorated SWCNTs are then dispersed in distilled deionized (dd) water (0.1 mg Pd-SWCNTs in 10 mL dd water). The solution is sonicated and successively drop-deposited onto the inter-digitated fingers to create a sensor with the initial resistance in the value of 1 kΩ associated with a zero concentration of methane.

**Figure 5.**
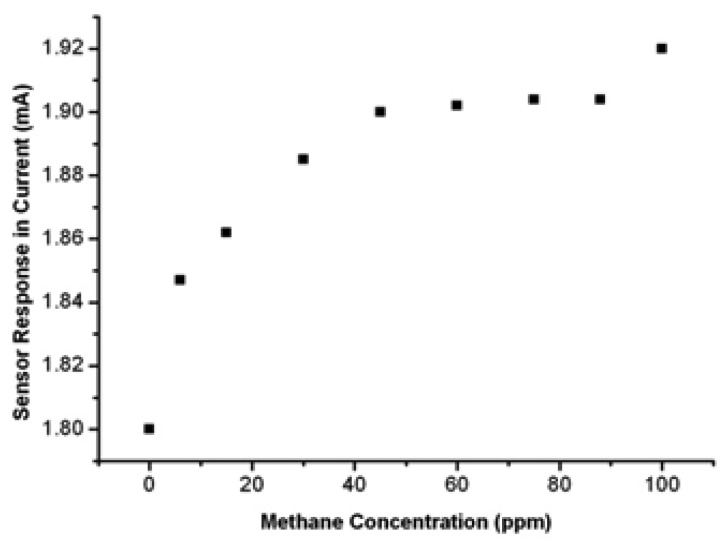
Current response *versus* concentration of methane.

Test results demonstrate an increase in current in the presence of small concentrations of methane. Figure 5 shows a typical current response curve, where we noticing that the current increases from 1.84 mA to 1.91 mA when the methane exposure increases from 6 ppm to 100 ppm at a constant value of 1 Volt applied on the IDE. In the same case, the typical resistance response will show a decrease from 0.543 kΩ and 0.523 kΩ.

### 3.3. RF/DC Rectifier

The RF/DC rectifier is a three-staged rectifier circuit assembled using a 0.5 μm CMOS process as proposed by Mandal *et al.* [27]. The frequency response of the proposed RF/DC rectifier is a band pass type, revealing a cutoff frequency at low band, a roll-off frequency at high band, and a DC voltage cross pass band. In terms of the UHF band in the RFID that is from 860 MHz to 960 MHz, the RF/DC rectifier outputs a stable and sustainable 1 V DC. When be directly wire-bonded to a printed circuit, the RF/DC rectifier is equivalent to *C_R_*, a capacitor of 0.27 pF. Due to the high sheet resistance of the polysilicon used in the transistor gates and capacitor plates in the process of the rectifier, a relatively large resistive component is achieved that increases the power-up threshold. When the input quality factor, *i.e.*, *Q_LO_* is 15.9, the rectifier is equivalent to *R_R_*, a parallel resistor of 10.3 kΩ.

## 4. Schematics of the Sensor Tag

**Figure 6.**
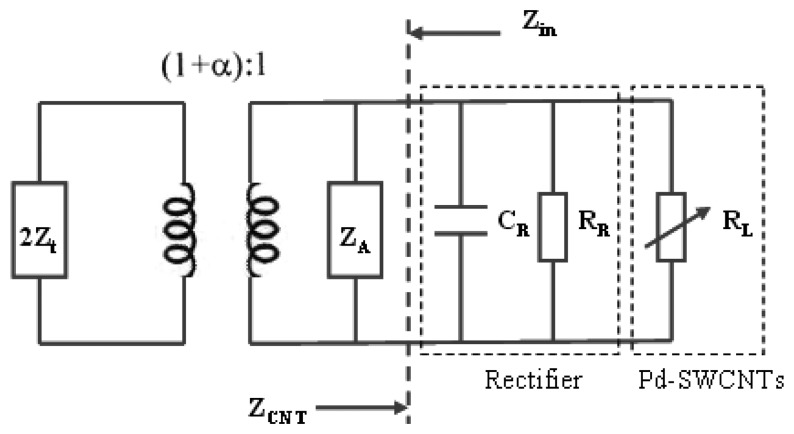
Schematic of the Sensor Tag.

Figure 6 show the equivalent circuit of the proposed sensor tag. *Z_CNT_* represents the input impedance toward the antenna load module that deals with the RF/DC rectifier's equivalent capacitor *C_R_*; the RF/DC rectifier's equivalent resistor *R_R_*; and the sensor head's equivalent resistor *R_L_*:

(7) $$Z_{\text{CNT}}=\frac{1}{R_{L}}+\frac{1}{R_{R}}+j\omega C_{R}$$

Provided the power delivered from the antenna module to the antenna load module reaches the maximum at the beginning of detection, *Z_CNT_* will be equal to *Z_in_*, the input impedance toward the antenna module. For the RF/DC rectifier, the equivalent capacitance *C_R_* is 0.27 pF, the equivalent resistance *R_R_* is 10.3 kΩ. At the beginning of the detection event the concentration of methane is zero, so the equivalent resistance of the sensor head *R_L_* is 1 kΩ.

According to the Equation (7), *Z_CNT_* is (300-*j*459) Ω that is equal to *Z_in_* equal to (300 + *j*459) Ω. We can figure out the exact dimension of the dipole and the T impedance matching networks from this. We also employ the finite differentiation of the time domain (FDTD) and the particle swarm algorithm to optimize this structure. The optimal size of the dipole and the T impedance matching network are listed in Table 1.

**Figure 7.**
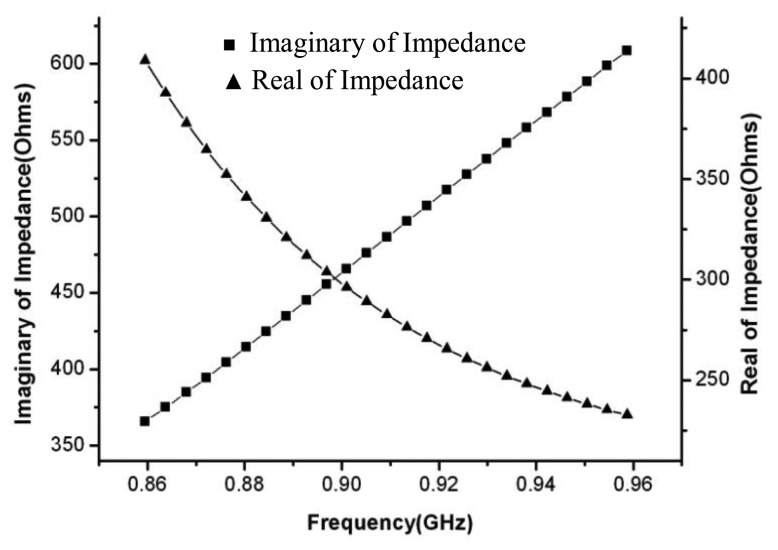
Input impedance of the antenna module *versus* frequency.

Figure 7 plots the real part and the imaginary part of the input impedance toward the antenna module. In the band of interest, the value of the real part and the imaginary part are closely equal to these that are strictly required by the input impedance toward the antenna load module. The results supply a design reference for the case that the dipole and the T impedance matching network are sized by *l* = *λ*/*2*, *w* = *λ*/200, *w′* = *λ*/200, *w′* = *w*/8, *Z_A_* = 75 Ω, in which, *Z_A_* is the dipole impedance taken at its center in the absence of a T-matching connection [28].

## 5. Characterization of the Sensor Tag

**Figure 8.**
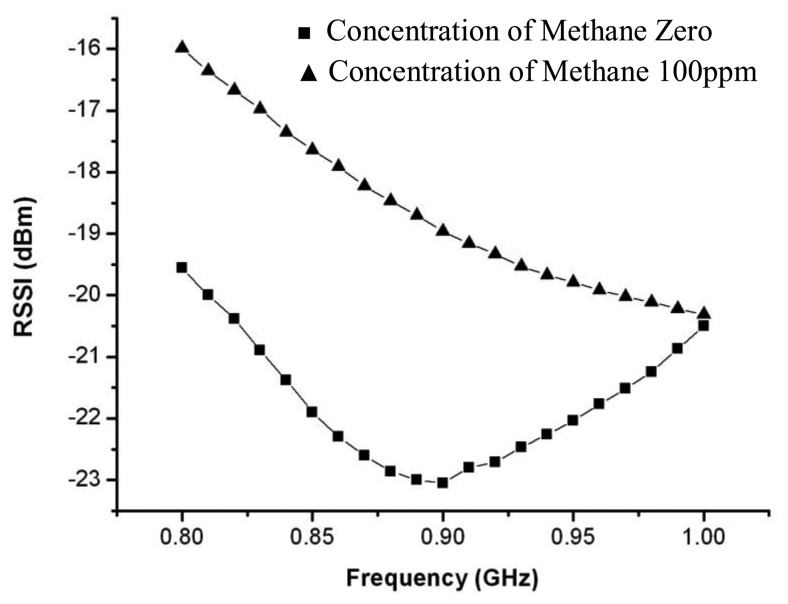
Variation of RSSI *versus* frequency with methane concentrations in ambient air.

The characterization of the proposed sensor tag is examined under a given scenario where the reader device is placed 1 meter away from the sensor tag. The reader device employs a typical dipole antenna that provides an omni-directional pattern in the horizon plane with a gain of 2.15 dBi. The power *P_in_* that the reader device sends out is 2 Watt. Figure 8 shows the variation of RSSI *versus* frequency under a given concentration level of methane in ambient air. At 900 MHz, the RSSI moves up from −23 dBm to −19 dBm, achieving a 4 dB total increase gap in response to the concentration of methane that increases from zero to 100 ppm.

Given *P^to^* denoting the minimum *P_in_* required by the RF/DC rectifier to put out 1 V DC, the input impedance toward the antenna module is matched with the input impedance of the antenna load module, it means:

(8) $$P^{to}(\varphi )={\left(\frac{\lambda_{0}}{4\pi r}\right)}^{-2}\frac{P_{\text{CNT}}}{G_{R}\;(\theta ,\phi )G_{T}\;(\theta ,\phi )\tau \;[\varphi (t)]}$$

In order to remove the influence caused by the gain of the antenna in the reader device, the gain of the antenna in the sensor tag, and the distance between the reader device and the sensor tag, is substituted into the expression in order to combine *P_CNT_* and *P_IC_* [29,30]. A non-dimensional indicator, denoted as the analog identifier (AID) can be introduced, that is:

(9) $$\text{AID}\;[\varphi (t)]=\frac{P_{\text{CNT}}}{2\sqrt{P_{IC}(\varphi )P^{to}}}=R_{\text{CNT}}\;[\varphi (t)]{\left|Z_{\text{CNT}}\;[\varphi (t)]+Z_{in}\right|}^{-1}$$

**Figure 9.**
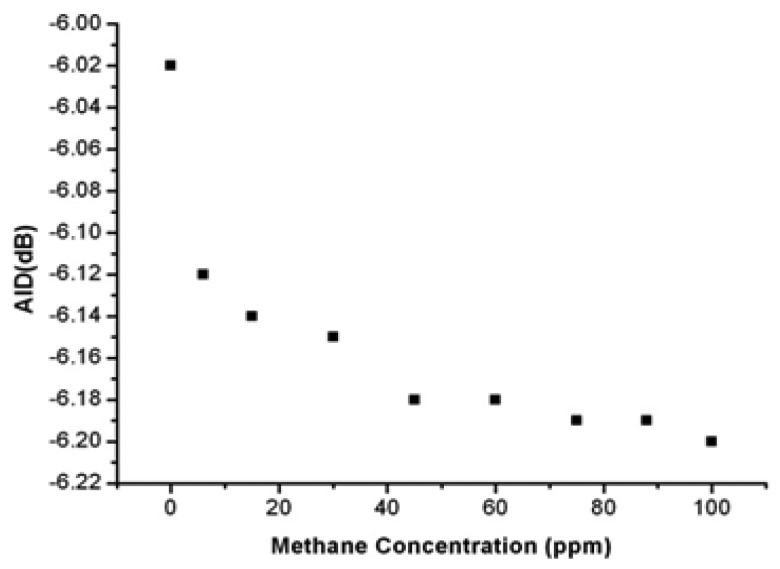
AID *versus* concentration of methane.

The AID factor, in fact, just depends on the impedance mismatch and is immune to the interrogation modalities. The AID parameter is examined under the same scenario where the methane concentration is varied from zero to 100 ppm. Supposing t_0_ represents the beginning of a specific detection event, the concentration of methane is zero, the AID(t_0_) is -6.02 dB. At the moment t_1_, when the concentration of methane is 100 ppm, the AID(t_1_) is −6.20 dB, as shown in Figure 9.

If the relative AID, *i.e.*, ΔAID is expressed by:

(10) $$\Delta \text{AID}=\frac{\text{AID}(t_{1})-\text{AID}(t_{0})}{\text{AID}(t_{0})}\cdot 100\%$$

It can be concluded that, in response to an increase in the concentration of methane from zero to 100 ppm, the ΔAID will display an increase by 3%.

## 6. Conclusions/Outlook

According to the design and the study of the proposed remote sensor for detecting the concentration of methane in room temperature, the following conclusions can be made:

(1) Pd-decorated SWCNTs have some sensitivity towards methane molecules in room temperature air. When used for fabricating a sensor, the Pd-SWCNTs will be the load of fingers dispensed on an IDE platform.
(2) The prerequisite for Pd-decorated SWCNTs to detect methane at room temperature is to supply a stable DC voltage onto the IDE electrodes. The required DC power can only be achieved from the tag's antenna; a high quality RF/DC rectifier is therefore indispensable.
(3) The antenna is the key part of the remote sensor because it carries out multiple functions including power collection, impedance matching and signal backscattering. The dipole plus T impedance matching network is the simplest but an effective scheme that can be a design paradigm helpful for the fabrication of different remote sensors.
(4) The RSSI and ΔAID are examined under a given scenario, showing an increase by 4 dB and by 3% with respect to a methane concentration variation from zero to 100 ppm. Although the parameters prove the ability of the proposed remote sensor to detect methane at room temperature, the recognizability is not big enough to give more accurate identification. Therefore, the study of more advanced Pd-decorated SWCNTs with enhanced recognition ability; the development of a more efficient RF/DC rectifier with a more stable voltage output, and a breakthrough in innovative antennas tailored for the RFID based sensor tag with a sound performance to complexity ratio are expected.

## Figures and Tables

**Table 1.** Size of dipole and T impedance match networks.

**Symbol**	***l***	***b***	***a***	***w***	***w*'**
size (mm)	175.00	3.05	87.00	1.68	0.20
